# Diseased Meats as Food

**Published:** 1893-02

**Authors:** Claude D. Morris


### DISEASED MEATS AS FOOD.

By Claude D. Morris, V.S.

There has been some literature of late on the question of diseased meats as an article of diet, and from all the results so far obtained through investigations, one is led to believe that the question is still an open one. It would seem that there could be no question, or any foundation for an argument. which had as its basis that animal flesh, slightly or otherwise tainted with disease, is not altogether unfit as food. And yet it is held by some, who occupy prominent positions in the field of science, that in this world, where there is so much want that scarce a year goes by but that some nation in distress is heard knocking at the door of a favored and prosperous nation, beseeching for the crumbs that fall from the bountifully-spread table of its commonwealth to feed their famishing children upon. An article of food so potential for the maintenance of life as animal flesh should not be ruthlessly wasted, because, perchance, some "crank" might think it unfit as food and have it consigned to the rendering establishment, while hundreds are in crying need of this apparent waste. It cannot be an unjust denial to withhold from those about us, who are less able to judge of the fitness of things, as an article of food which science or the result of investigation may show to be hurtful as such, no more than it would be unjust to refuse a child that which would prove a deadly poison, simply because the child should want it. It has taken centuries for man to prove that certain plants in the vegetable kingdom are wholesome and nutritious as food. It has cost the lives of many investigators to establish the usefulness of herbs as to their materia medica, and it is upon this information that the people of this generation are enjoying the beneficence of those who have sacrificed for their good.

We must accept the results of their labor and be willing to be led by the guiding hand of progress, the inevitable of all ages, evolution.

The real progress and wealth of a nation is made manifest by the character of its people. And man's ability to progress evolves as the result of his daily habits in the exercise of his mental and corporeal energies.

The practice of medicine teaches that there exists an intimate relation between the health of man and his use of flesh as food. An albuminous diet creates energy, and energy develops all the latent powers, and there being no one article of dietary which contains so great an amount of proteids as flesh, it, therefore, becomes a matter of national economy and an indirect civilizing force. Thus it is of the utmost importance that the people of this nation, who consume by far the greatest quantity of animal flesh per capita of any nation on the globe, should be restricted to that which is wholesome and free from diseases.

While wholesome meats are of the greatest importance in the maintenance of health, on the other hand, diseased meats without doubt environ society with many dangers.

It is needless to reiterate here the experiments and their results which have been undertaken of late to prove that tuberculosis is a contagious disease, not only by contact, but through alimentation also. As readers of the veterinary publications of this country are more or less familiar with the character of current thought on this subject, and what is true of the menace to society arising from one contagious disease is more or less affirmative in its relation to other contagious diseases.

An animal suffering with a contagious malady is a menace to health, and if the products of such animals are used as food it becomes a greater source of danger. These are the conclusions of the ablest writers on sanitation. This was the conclusion of the recent Veterinary Congress held at Paris, which convened for the purpose of unfolding the truth regarding contagious disease among the lower animals and their relations to man.

Within the past few years there has developed in New York City a demand for cheap meats of a very inferior quality. This demand is on the increase.

It has only been recently that the farmer, living in the vicinity of the great metropolis, could hope to find a market for the old, worn-out, thin in flesh and diseased animals of his herd. But to-day the rural owner of such cattle finds a ready market and fair prices for this grade of meat cattle. The Union Stock Yards is the unloading place for nearly all the animals which are intended for slaughter in New York City.

There is no doubt but that some of the finest grades of fat cattle grown in this country pass through these yards. And there is less doubt but that some of the worst diseased and worn-out cattle pass these same yards on their march to the consumer's table. I shall speak of cows only in this relation. There are about two hundred and fifty animals each week arriving at these yards, about one-fourth of which are fresh milch cows. The milch cows, as a rule, are healthy, vigorous-looking animals, and many of those intended for slaughter are in good health, but in the majority of cases are thin in flesh; but out of these comes a class that should not be allowed for consumption. In some of the recently published articles on meat inspection it has been shown that sanitary precaution in this work is very old.

Implying that the Jews, centuries ago, passed rigid laws relative to the inspection of meats, and that for this reason there was no class of people better qualified to determine as to the fitness of meats for consumption than the Jew.

There is no question but that the early or primeval Jew was the sanitarian of his day, but that was three and four thousand years ago. However that may be, at the present time his ecclesiastical sanitation has given place to his more American idea of money making. Strange as this fact may seem, all the abattoirs of New York and Brooklyn are under the control of Jews, and nine-tenths of all the meat markets in these cities are under the same supervision. The writer has seen this sanitarian Jew, who was delegated by the church to cut the throats of animals at the slaughter house and pass judgment upon their fitness as food, take to his home an unborn calf of from five to six months in utero, as it was relieved from its mother's womb on the dressing bed, and prepare the same for food for himself and family. I do not say that this sort of diet is absolutely dangerous, but I fear my search would be in vain were I looking for an American-born citizen who would eat such food. It is the foreign-born population of New York City that has created a demand for cheap and unwholesome meats. I would not condemn meat as being unfit for food that was thin in flesh only. It is against diseased meats that I would sound an alarm.

I will submit a few facts in relation to existing conditions at one of the abattoirs of New York City. And what is true of this one is true to a greater or less degree of others that have come under my observation. From July 1st, 1892, to January 1st, 1893, there were slaughtered in this abattoir 3,601 cows. Out of this number 492 were sick; 237 were sick with contagious disease, and 255 were sick with non-contagious disease; 15 were at the point of death, as the consequence of their malady, when driven into the shoots to be slaughtered, and 3 were dressed and hung up that had died on the street en route to the abattoir.

Of this number of 492 sick animals, 209 had tuberculosis, represented in its varying stages, from the small pearly-like tubercle, as seen on the serous membrane, to complete caseation and calcification of the tissues involved, and in some instances the carcass would give forth a very offensive odor; 134 had pleurisy associated with its complications, 121 had mammatis of the entire udder, and in the majority of cases to the extent of profuse suppuration; 18 had Texas fever, and had these been left to themselves a few hours longer they would have succumbed, and as before stated, 3 died en route to the slaughter house, and 10 had actinomycosis. Out of this total of 492 diseased carcasses, not one of which was fit for food, the municipal inspection of slaughtered meats consigned only 9 to the offal, that being done in a perfunctory way, and by no means the worst ones were cut down.

There is yet much material for thought by following the product of this sanitarian Jew's slaughter house to its final destination. It goes to the occupants of the thickly packed houses of foreign-born population in New York City. In the first place, this grade of meat is handled by Hungarians and Polish Jews. The Russian Jew is in the market to a certain extent; their market places are mostly in basements, illy ventilated and filthy. It is a disgusting sight to visit one of these markets. I visited a few of the tenements where these meats are consumed, and to say the most in their favor, these apartments were not at all inviting; they were, in every sense, fit hotbeds for contagion.

Investigation into the origin of Asiatic cholera, shows that it is a disease, primarily, of the unclean and filthy classes. Typhus fever, that has developed in certain localities in New York City during this season, has been found, in every instance, to have its origin in dirty tenements or cheap boarding houses. These are the dreaded diseases of our time. And who are the people that are generally attacked with these malignant maladies? Universally the foreigner.

These are foreign diseases to this country. It therefore becomes a matter of more than municipal concern, if in the great metropolis of this country existing circumstances are such that it becomes a laboratory for the propagation of contagion and infection, both domestic and foreign.

What are the conditions favorable to cultivate and spread contagion? Foul cloths, unclean bodies, polluted atmosphere, and diseased meats, when consumed as food. And these are the exact conditions which exist among a large portion of the foreign-born population in New York City.

These habits and mode of living are unnatural in this country. They are the customs belonging to the foreigner who brings them with him as he comes to our shores. In the face of existing facts, that there is a tendency on the part of foreigners to perpetuate the customs which have become the established character of portions of darker Europe, whose daily life has not been one of progression, and who are, many of them, nothing more nor less than the vicious and criminal classes of their native land, upon arriving here cannot, in the nature of things, possibly appreciate the conditions of American freedom, much less assume the responsibilities of citizenship, is in its self a source of infection and contagion in this country. And this is the class who live in colonies in these large cities, whose mode of living invite disease.

I do not desire to criticise unjustly this great mass of foreign-born population, many of whom come to our shores to vow allegiance to our form of government. These are industrious, frugal and honest. They are found engaged in all the pursuits of industry, and they seize those opportunities with all the zeal and patriotism of loyal Americans, and to such the beneficent arm of our great commonwealth is always extended. But this class is not the whole of emigration. In this paper I have not attempted to give any facts relative to the evil effects of unwholesome food. I hope to do so, however, in a subsequent paper.

What are the measures necessary to eradicate the sale of diseased and unwholesome meats ? There is but one way to make it effective, and that is through federal inspection. A thorough inspection of all cattle previous to being slaughtered, and a thorough post-mortem. With public abattoirs erected upon the principles of sanitation, centralized, and under rigid restrictions, with veterinarians for inspectors whose qualifications and integrity are known.

